# Supplementary material for: Single-cell analysis of uterosacral ligament revealed cellular heterogeneity in women with pelvic organ prolapse
Source: Commun Biol. 2024 Feb 7;7:159. doi: 10.1038/s42003-024-05808-3 (PMC10850063; doi:10.1038/s42003-024-05808-3)
Supplement: Supplementary file 2 — Supplementary Figure [file 42003_2024_5808_MOESM2_ESM.pdf]

Supplementary Figure 1

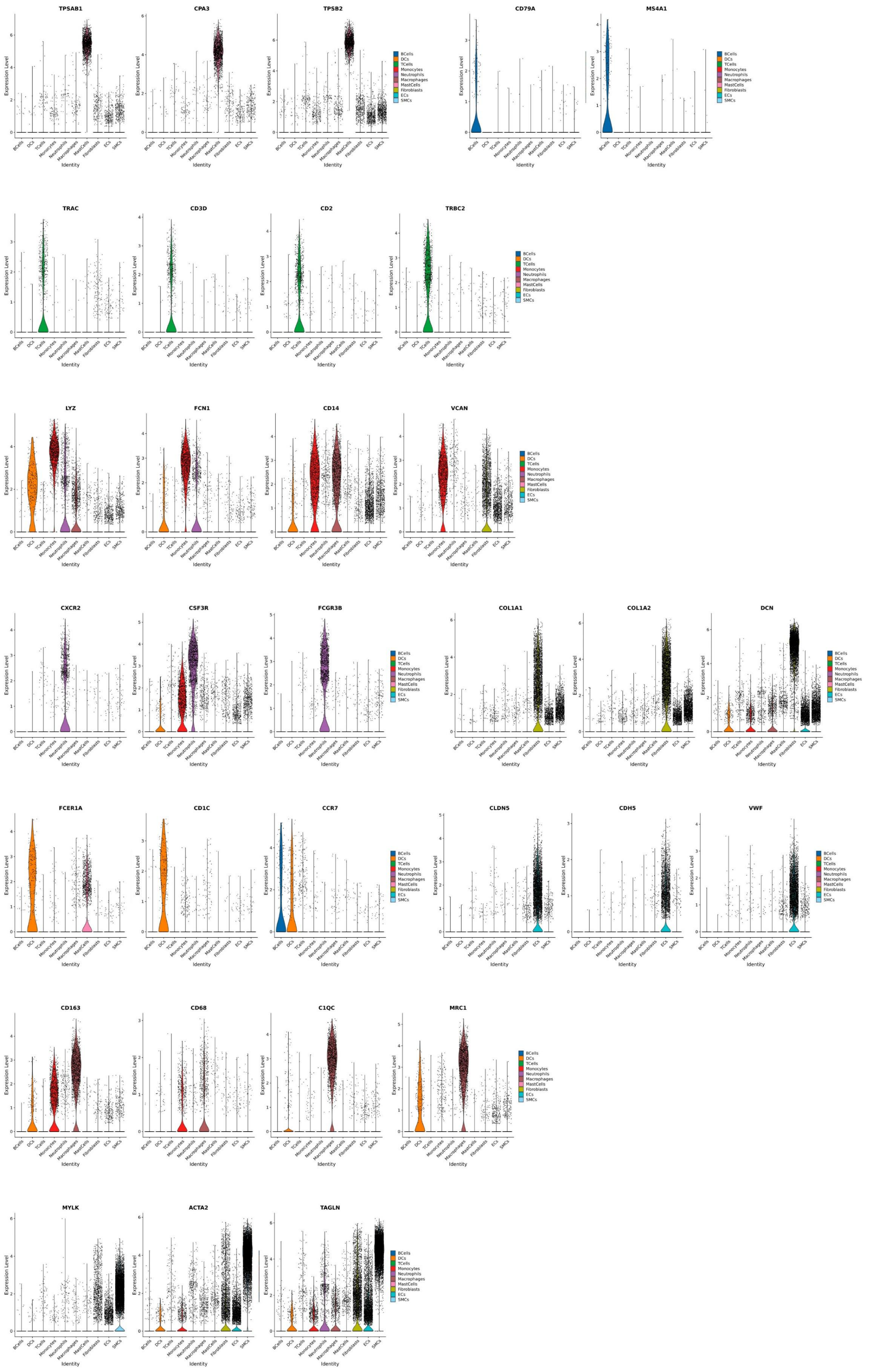

Violin plots showing the expression of canonical markers for each cell type.

Supplementary Figure 2

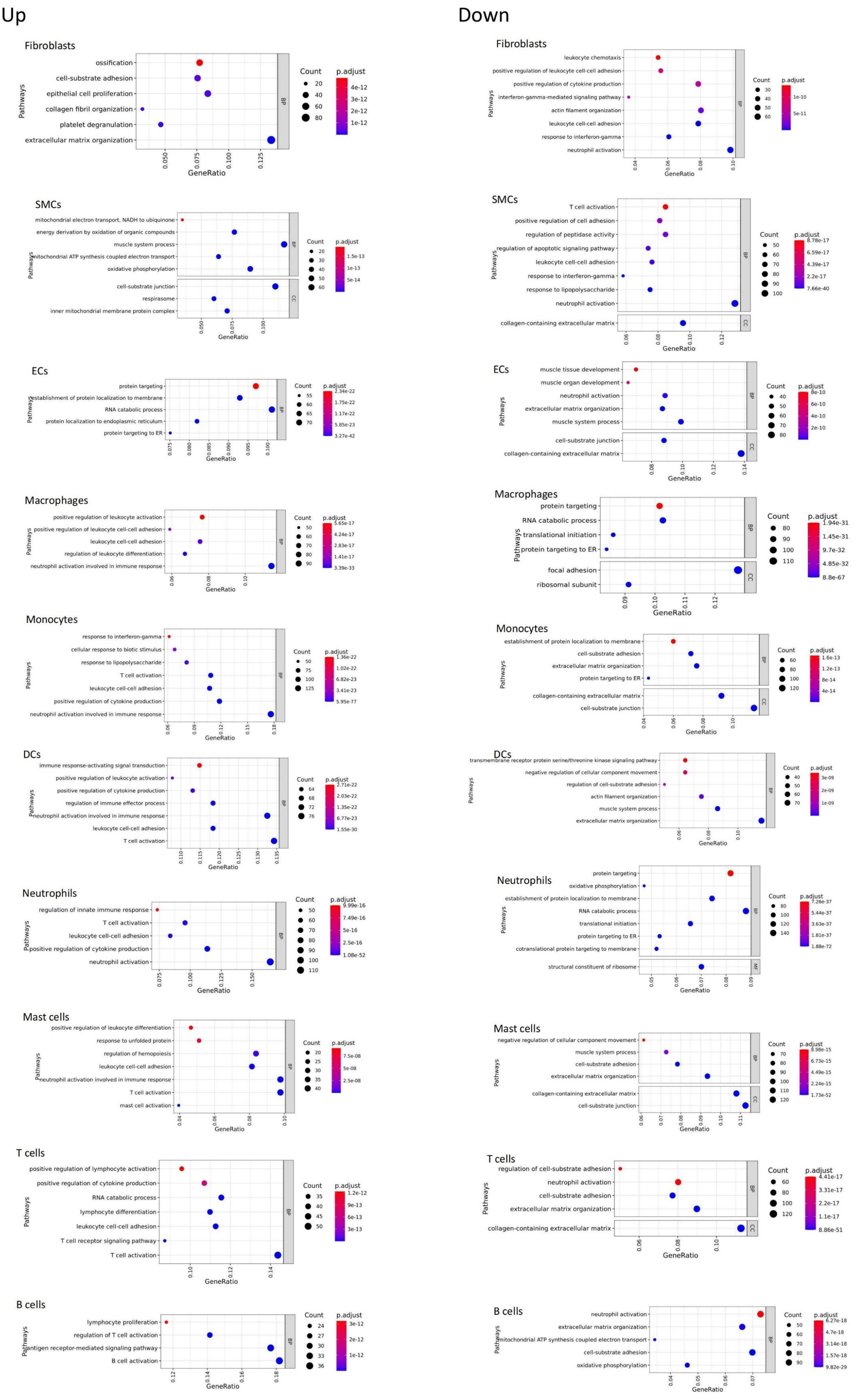

Dot plots displaying the GO enrichment of up- or downregulated genes of each cell type in POP and control samples.

Supplementary Figure 3

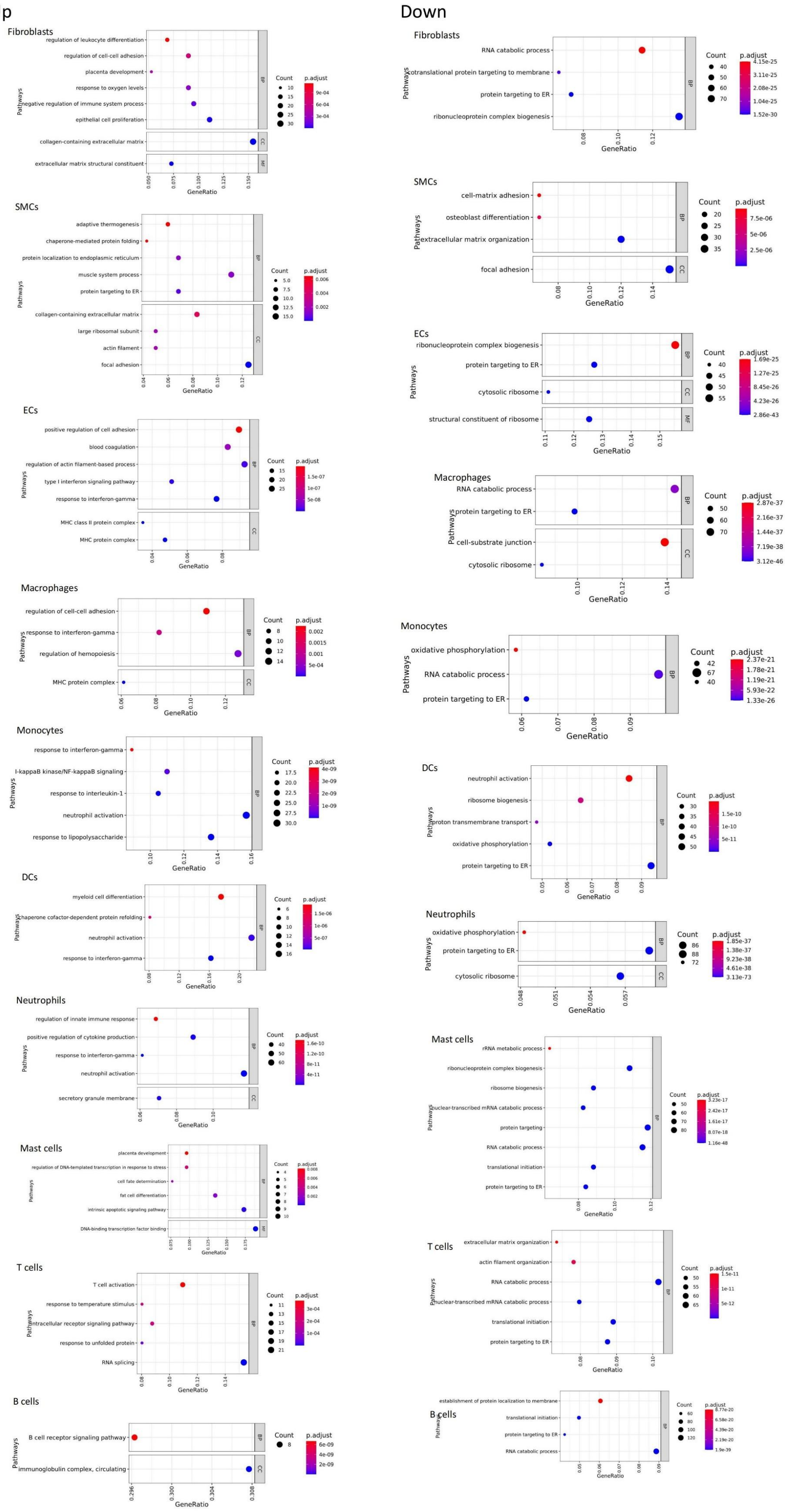

Dot plots displaying GO enrichment of up- or downregulated genes of each cell type in POP group compared with the control group.

Supplementary Figure 4

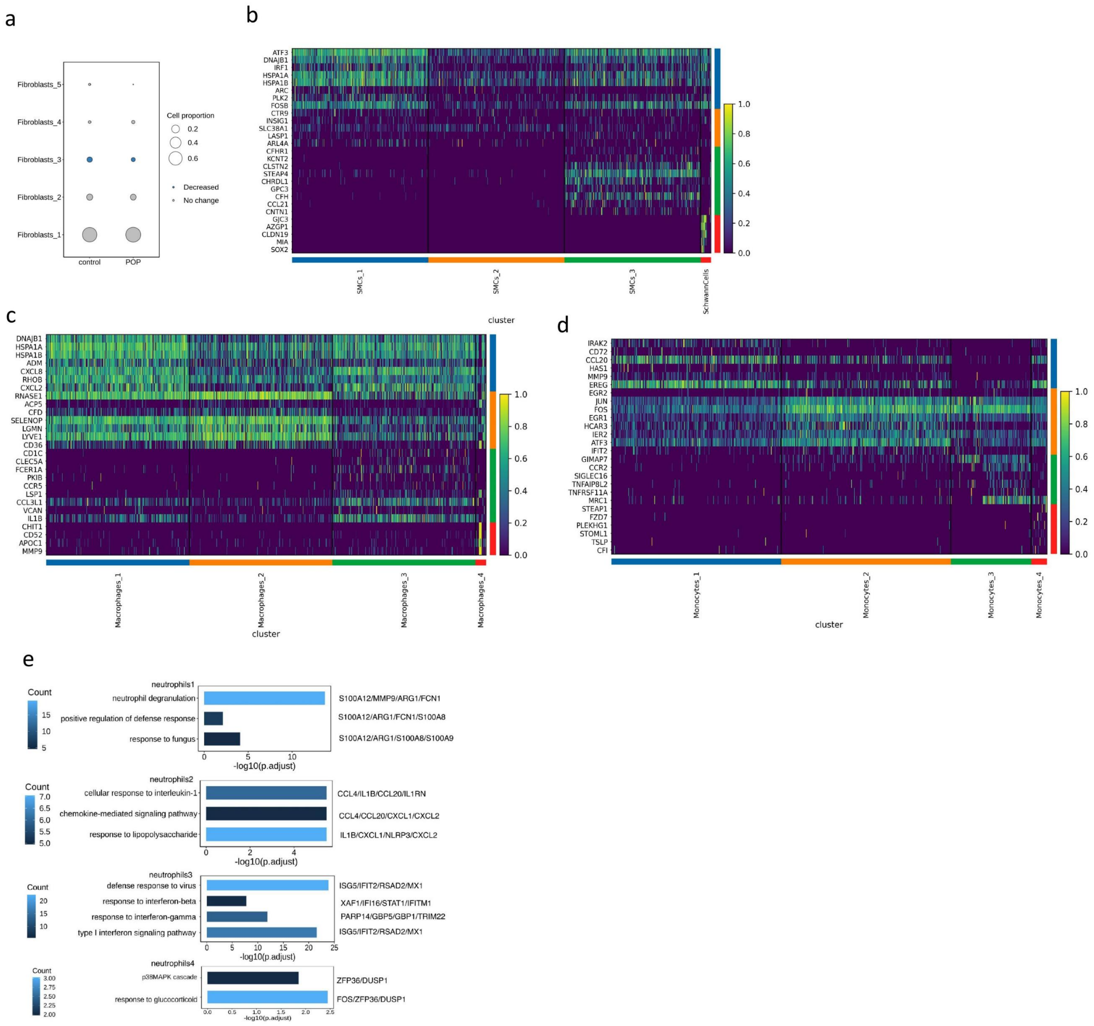

(a) Dot plot displaying relative changes in cell ratios of the subclusters of fibroblasts across control and POP samples.

(b) Heatmap showing the enriched genes of four SMC subclusters.

(c) Heatmap showing the enriched genes of four macrophages subclusters.

(d) Heatmap showing the enriched genes of four monocytes subclusters.

(e) Bar plots displaying GO enrichment of upregulated genes of four neutrophils subclusters in USL samples.

Supplementary Figure 5

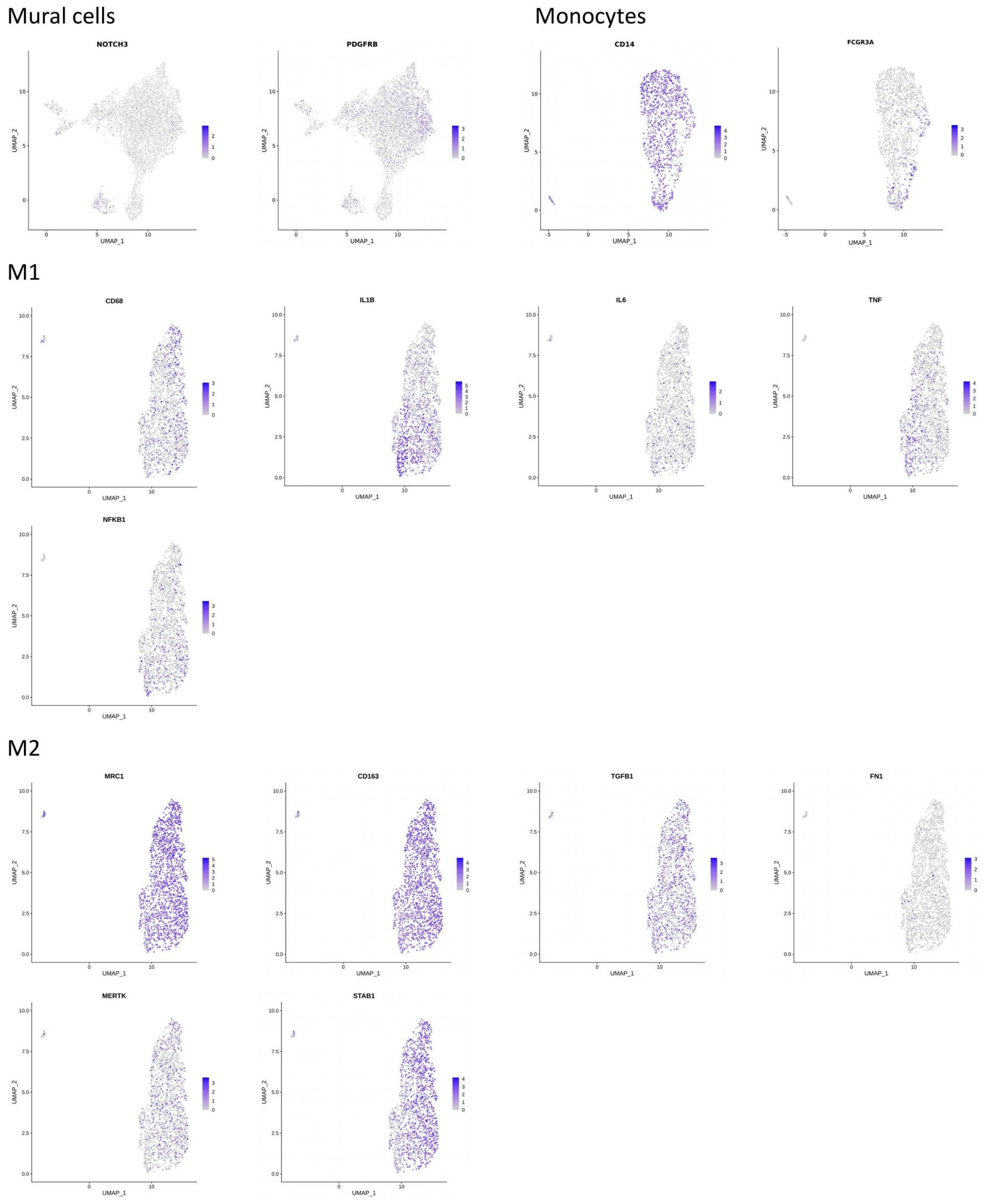

UMAP plots showing expression of canonic markers for mural cells, monocytes subtypes, M1 and M2 cells.

Supplementary Figure 6

a

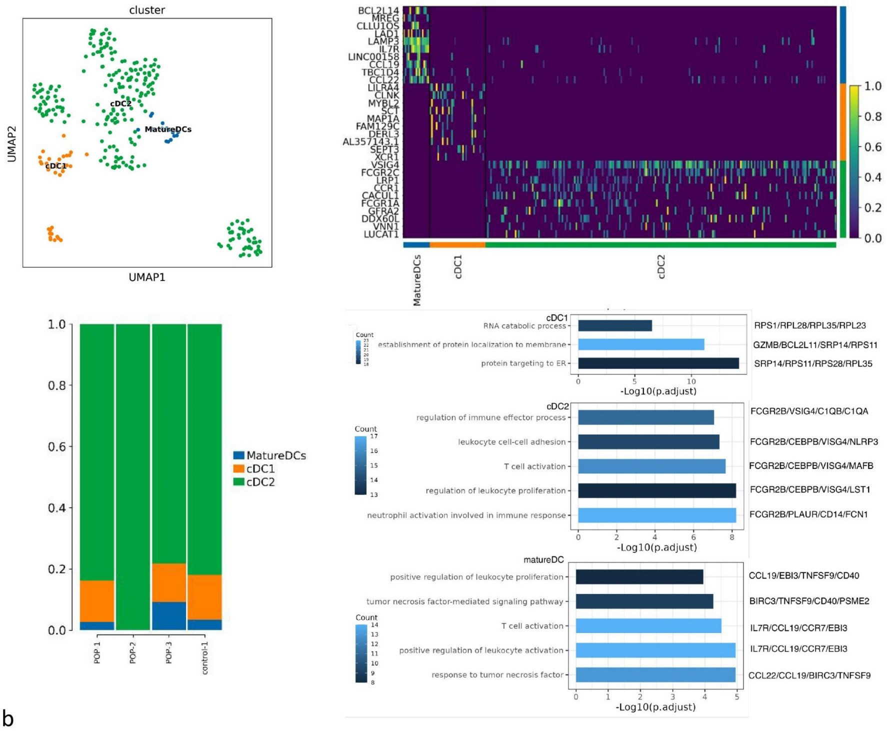

b

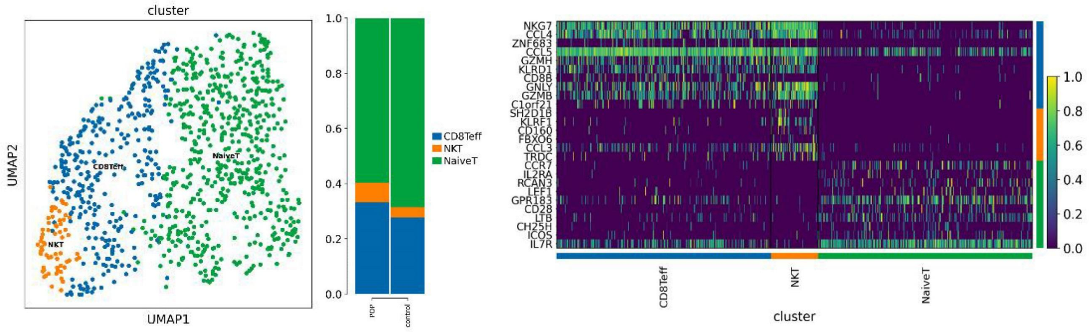

Subclustering of DCs and T cells in USL.

(a) UMAP plot showing the subclusters of DCs. Two bar plots showing the percentage and GO enrichment of upregulated genes of three DC subclusters in POP and control samples. Heatmaps showing top 10 differentially expressed genes of three DC subclusters respectively.

(b) UMAP plot showing the subclusters of T cells. Bar plot showing the percentage and heatmap showing top 10 enriched genes of three T cell subclusters.

Supplementary Figure 7

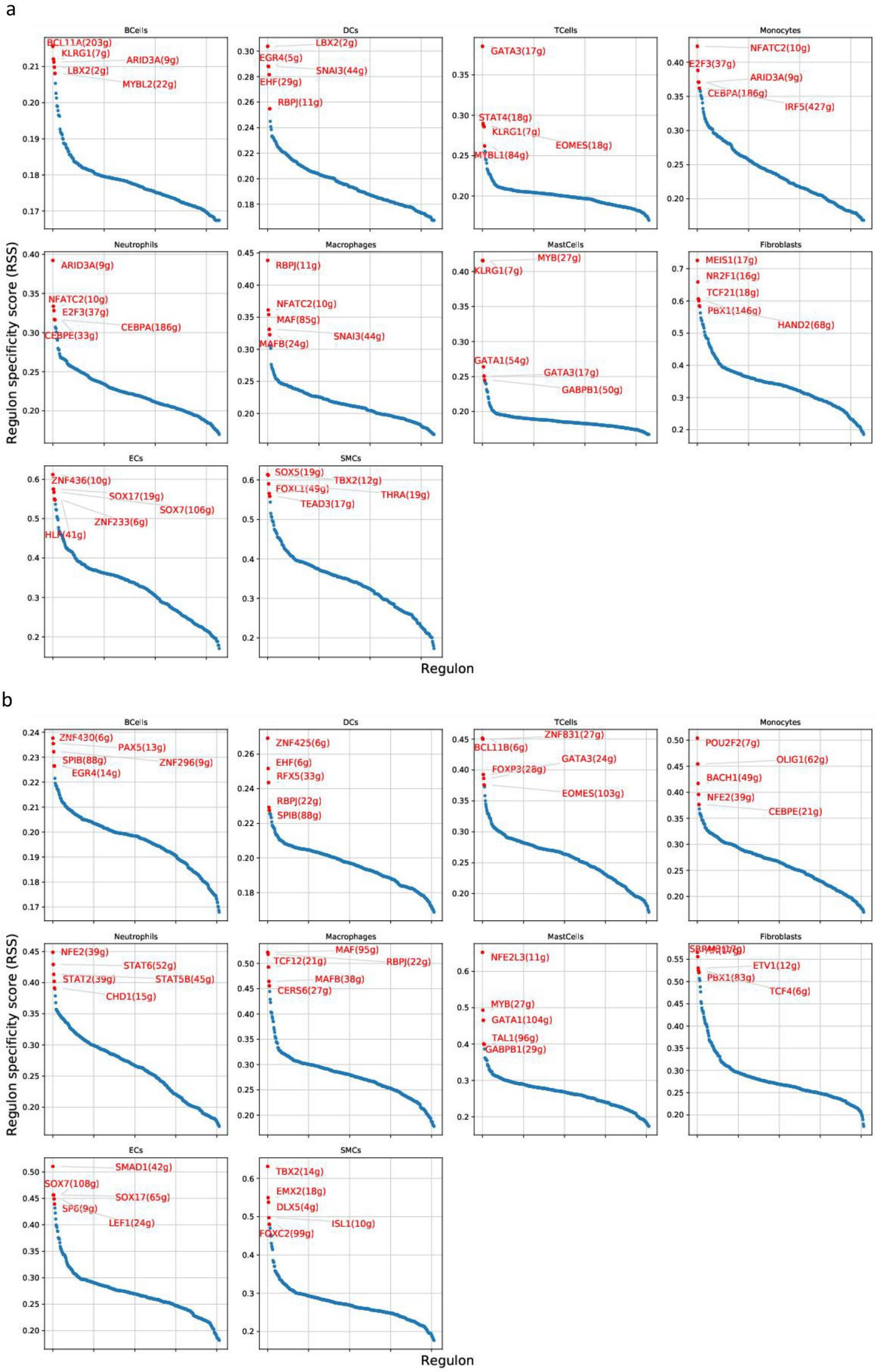

The Scatter plots showing the top 5 cell type-specific TFs.

(a)The scatter plot showing the top 5 cell type-specific TFs in each cell type in control sample.

(b)The scatter plot showing the top 5 cell type-specific TFs in each cell type in POP samples.
