# Supplementary material for: Single-cell analysis of uterosacral ligament revealed cellular heterogeneity in women with pelvic organ prolapse
Source: Commun Biol. 2024 Feb 7;7:159. doi: 10.1038/s42003-024-05808-3 (PMC10850063; doi:10.1038/s42003-024-05808-3)
Supplement: Supplementary file 3 — Description of Additional Supplementary Files [file 42003_2024_5808_MOESM3_ESM.docx]

**Description of Additional Supplementary Files**

**File name:** Supplementary Data 1

**Description:**

Sheet 1: Clinical characteristics of POP and control patients profiled by sc-RNA seq in this study.

Sheet 2: The source data of Figure 1d (The cell number of each population in each sample)

Sheet 3: The source data of Figure 2c, 3b, 3h, 4b, 4f, 5f (The cell number of each subpopulation in each sample)

Sheet 4: The source data of Figure 2d

Sheet 5: The source data of Figure 3d

Sheet 6: The source data of Figure 3j

Sheet 7: The source data of Figure 4d

Sheet 8: The source data of Figure 4h

Sheet 9: The source data of Figure 5i

Sheet 10: The source data of Figure1c

Sheet 11: The source data of Supplementary Figure 1

**File name:** Supplementary Data 2

**Description:**

Sheet 1: The source data of Figure 6e

Sheet 2: The source data of Figure 6f

Sheet 3: The source data of Figure 6g

Sheet 4: The source data of Figure 6h

Sheet 5: The source data of Figure 6i

Sheet 6: The source data of Figure 7b

Sheet 7: The source data of Figure 7d

Supplementary Information: Supplementary Figure
