# Supplementary material for: Single-cell analysis of uterosacral ligament revealed cellular heterogeneity in women with pelvic organ prolapse
Source: Commun Biol. 2024 Feb 7;7:159. doi: 10.1038/s42003-024-05808-3 (PMC10850063; doi:10.1038/s42003-024-05808-3)
Supplement: Supplementary file 6 — Reporting Summary [file 42003_2024_5808_MOESM6_ESM.pdf]

nature portfolio

Corresponding author(s): Xiaochun Liu

Last updated by author(s): Jan 1, 2024

Reporting Summary

Nature Portfolio wishes to improve the reproducibility of the work that we publish. This form provides structure for consistency and transparency in reporting. For further information on Nature Portfolio policies, see our Editorial Policies and the Editorial Policy Checklist.

Statistics

For all statistical analyses, confirm that the following items are present in the figure legend, table legend, main text, or Methods section.

n/a	Confirmed	
☐	☒	The exact sample size (n) for each experimental group/condition, given as a discrete number and unit of measurement
☐	☒	A statement on whether measurements were taken from distinct samples or whether the same sample was measured repeatedly
☐	☒	The statistical test(s) used AND whether they are one- or two-sided<br>*Only common tests should be described solely by name; describe more complex techniques in the Methods section.*
☒	☐	A description of all covariates tested
☐	☒	A description of any assumptions or corrections, such as tests of normality and adjustment for multiple comparisons
☐	☒	A full description of the statistical parameters including central tendency (e.g. means) or other basic estimates (e.g. regression coefficient) AND variation (e.g. standard deviation) or associated estimates of uncertainty (e.g. confidence intervals)
☐	☒	For null hypothesis testing, the test statistic (e.g. F, t, r) with confidence intervals, effect sizes, degrees of freedom and P value noted<br>*Give P values as exact values whenever suitable.*
☒	☐	For Bayesian analysis, information on the choice of priors and Markov chain Monte Carlo settings
☒	☐	For hierarchical and complex designs, identification of the appropriate level for tests and full reporting of outcomes
☒	☐	Estimates of effect sizes (e.g. Cohen's d, Pearson's r), indicating how they were calculated

Our web collection on statistics for biologists contains articles on many of the points above.

Software and code

Policy information about availability of computer code

Data collection	Poly-A tails and adaptor sequences were removed by cutadapt v3.7. After quality control, reads were mapped to the reference genome GRCh38 (ensembl version 92 annotation) using STAR v2.6.1a. Gene counts and UMI counts were acquired by featureCounts v2.0.1 software. Expression matrix files for subsequent analyses were generated based on gene counts and UMI counts.
Data analysis	For data processing and analysis, Scanpy (v1.8.2), Seurat (v3.1.2), clusterProfiler (v3.16.1), CellPhoneDB (v2.1.0), Monocle2 (v 2.22.0), velocyto(v 0.2.3), scVelo (v0.17.17), pyscenic (v0.11.0) are used under R (v3.6.3) or python (v3.7).

For manuscripts utilizing custom algorithms or software that are central to the research but not yet described in published literature, software must be made available to editors and reviewers. We strongly encourage code deposition in a community repository (e.g. GitHub). See the Nature Portfolio guidelines for submitting code & software for further information.

Data

Policy information about availability of data

All manuscripts must include a data availability statement. This statement should provide the following information, where applicable:

- Accession codes, unique identifiers, or web links for publicly available datasets
- A description of any restrictions on data availability
- For clinical datasets or third party data, please ensure that the statement adheres to our policy

Our scRNA-seq data are accessible in Gene Expression Omnibus under the accession number GSE250414 which include the source data. Other data are available from the corresponding authors on reasonable request.

Research involving human participants, their data, or biological material

Policy information about studies with human participants or human data. See also policy information about sex, gender (identity/presentation), and sexual orientation and race, ethnicity and racism.

Reporting on sex and gender	All participants are female.
Reporting on race, ethnicity, or other socially relevant groupings	All participants are Asians and Han Chinese.
Population characteristics	All patients were non-smokers, aged >50 years, and had a BMI ranging 18.5–28 kg/m2.
Recruitment	This study included women who underwent hysterectomy for POP and hysteromyoma. Patients with Pelvic Organ Prolapse Quantification (POP-Q) stage III–IV were enrolled in the study following the diagnosis by the same experienced specialist. Women who underwent hysterectomy for hysteromyoma and did not have prolapse were included as the control group.
Ethics oversight	This study was approved by the Ethics Committee of Shanxi Bethune Hospital (YXLL-2022-131).

Note that full information on the approval of the study protocol must also be provided in the manuscript.

Field-specific reporting

Please select the one below that is the best fit for your research. If you are not sure, read the appropriate sections before making your selection.

☒ Life sciences ☐ Behavioural & social sciences ☐ Ecological, evolutionary & environmental sciences

For a reference copy of the document with all sections, see nature.com/documents/nr-reporting-summary-flat.pdf

Life sciences study design

All studies must disclose on these points even when the disclosure is negative.

Sample size	Approximately 1–1.5 cm3 of the cervical attachment of the USL was collected from the hysterectomy specimen of the patient
Data exclusions	Scanpy v1.8.2 was used for quality control, dimensionality reduction and clustering under Python 3.7 55. For each sample dataset, we filtered expression matrix by the following criteria: 1) cells with gene count less than 200 or with top 2% gene count were excluded; 2) cells with top 2% UMI count were excluded; 3) cells with mitochondrial content > 30 % were excluded; 4) genes expressed in less than 5 cells were excluded.
Replication	This study had no technical replicates.
Randomization	Patients were not randomized in this study because randomization is not relevant to our study design.
Blinding	There was no blinding in this study.

Reporting for specific materials, systems and methods

We require information from authors about some types of materials, experimental systems and methods used in many studies. Here, indicate whether each material, system or method listed is relevant to your study. If you are not sure if a list item applies to your research, read the appropriate section before selecting a response.

Materials & experimental systems

n/a	Involved in the study
☒	☐ Antibodies
☒	☐ Eukaryotic cell lines
☒	☐ Palaeontology and archaeology
☒	☐ Animals and other organisms
☒	☐ Clinical data
☒	☐ Dual use research of concern
☒	☐ Plants

Methods

n/a	Involved in the study
☒	☐ ChIP-seq
☒	☐ Flow cytometry
☒	☐ MRI-based neuroimaging

Plants

Seed stocks

Report on the source of all seed stocks or other plant material used. If applicable, state the seed stock centre and catalogue number. If plant specimens were collected from the field, describe the collection location, date and sampling procedures.

Novel plant genotypes

Describe the methods by which all novel plant genotypes were produced. This includes those generated by transgenic approaches, gene editing, chemical/radiation-based mutagenesis and hybridization. For transgenic lines, describe the transformation method, the number of independent lines analyzed and the generation upon which experiments were performed. For gene-edited lines, describe the editor used, the endogenous sequence targeted for editing, the targeting guide RNA sequence (if applicable) and how the editor was applied.

Authentication

Describe any authentication procedures for each seed stock used or novel genotype generated. Describe any experiments used to assess the effect of a mutation and, where applicable, how potential secondary effects (e.g. second site T-DNA insertions, mosiacism, off-target gene editing) were examined.
